# Severe Peripheral Arterial Ischemia Leading to Digital Gangrene in Mixed Connective Tissue Disease: A Case Report

**DOI:** 10.7759/cureus.106861

**Published:** 2026-04-11

**Authors:** Manuel Esaú Tamayo-Gómez, Alfonso Sandoval, Lilian Priscilla Arjona-Bojorquez, Luis Manuel Pablos-López, María Abril Mangas-Sosa, José Emiliano González Flores

**Affiliations:** 1 General Surgery, "Dr. Javier Buenfil Osorio" General Hospital of Specialties, Campeche, MEX; 2 Medicine, Tecnológico de Monterrey Campus Ciudad de Mexico, Mexico City, MEX; 3 Surgery, Autonomous University of Campeche, Campeche, MEX

**Keywords:** autoimmune vascular injury, digital gangrene, mixed connective tissue disease, peripheral ischemia, raynaud phenomenon.

## Abstract

Mixed connective tissue disease (MCTD) is a rare systemic autoimmune disorder characterized by overlapping clinical features of systemic lupus erythematosus, systemic sclerosis, and polymyositis, in association with anti-U1 ribonucleoprotein (anti-U1-RNP) antibodies. Although vascular manifestations such as Raynaud’s phenomenon are common, severe peripheral ischemic complications are rare. We report the case of a 40-year-old woman with known MCTD who presented with a three-month history of progressive necrotic lesions affecting the lower extremities, associated with severe pain, impaired ambulation, and ulcerative lesions of the hands. Her disease course was complicated by interstitial lung disease and pulmonary arterial hypertension. On admission, physical examination revealed extensive gangrenous lesions affecting multiple toes, along with cutaneous and musculoskeletal features consistent with advanced disease. Despite multidisciplinary management, the ischemic lesions progressed, and surgical intervention was planned. The patient subsequently developed sudden cardiorespiratory arrest and died before surgery.

This report highlights a rare and severe vascular manifestation of MCTD characterized by peripheral arterial ischemia leading to digital gangrene. Clinicians should maintain a high index of suspicion for systemic autoimmune diseases in patients presenting with unexplained ischemic or necrotic lesions. Early recognition and multidisciplinary management are essential to improve clinical outcomes.

## Introduction

Mixed connective tissue disease (MCTD) was first described by Sharp in 1972 as a systemic autoimmune disorder characterized by overlapping clinical features of systemic lupus erythematosus, systemic sclerosis, and polymyositis [1,2]. The disease predominantly affects women in the fourth decade of life and is considered a rare connective tissue disorder. Although its exact etiology remains unclear, MCTD is believed to result from a complex interaction between genetic susceptibility and immune dysregulation. Genetic associations involving HLA alleles, including HLA-B08 and HLA-DRB104:01, have been described as potential risk factors for disease development [2,3]. Current evidence suggests that autoreactive T and B lymphocytes contribute to the production of pathogenic autoantibodies that target endothelial and pulmonary tissues, while the recognition of ribonucleoproteins contained in apoptotic vesicles may also play a role in disease pathogenesis [2,3].

The clinical spectrum of MCTD is broad and may involve multiple organ systems, including musculoskeletal, pulmonary, cutaneous, and vascular systems. Among systemic complications, pulmonary involvement, particularly interstitial lung disease and pulmonary arterial hypertension, is a major determinant of morbidity and mortality [4]. Management strategies are largely extrapolated from treatments used for other connective tissue diseases and typically include glucocorticoids, antimalarial agents such as hydroxychloroquine, and immunosuppressive therapies based on the extent of organ involvement [5,6]. Vascular manifestations are frequently observed in patients with MCTD, most commonly in the form of Raynaud’s phenomenon; however, severe peripheral ischemic complications are rare and may indicate advanced immune-mediated vascular injury.

We report a case of severe peripheral arterial ischemia leading to digital gangrene in a patient with mixed connective tissue disease. This report aims to describe a rare, severe vascular manifestation of MCTD and to highlight the importance of early recognition of systemic autoimmune conditions in patients presenting with unexplained peripheral ischemia. Additionally, this case seeks to contribute to the existing literature by emphasizing the potential role of immune-mediated vascular injury in the development of severe ischemic complications in MCTD.

## Case presentation

A 40-year-old woman with a known history of MCTD presented to our institution with a three-month history of progressive necrotic lesions of the lower extremities associated with severe pain and impaired ambulation, as well as ulcerative lesions involving both hands. Three years before admission, the patient had initially sought medical evaluation due to progressive dyspnea on moderate exertion and episodes of Raynaud’s phenomenon. She had been evaluated by pulmonology and cardiology specialists, and diagnostic workup had revealed diffuse interstitial lung disease with a nonspecific interstitial pneumonia (NSIP) pattern, as well as pulmonary arterial hypertension. Physical examination at that time had also demonstrated sclerodactyly and clinical findings consistent with chronic inflammatory arthritis. Given the coexistence of interstitial lung disease and autoimmune manifestations, further rheumatologic evaluation had been performed. Serologic testing had revealed elevated titers of anti-U1 ribonucleoprotein (anti-U1-RNP) antibodies, supporting the diagnosis of MCTD. The patient had been subsequently treated with systemic corticosteroids and supplemental oxygen therapy.

**Figure 1:**
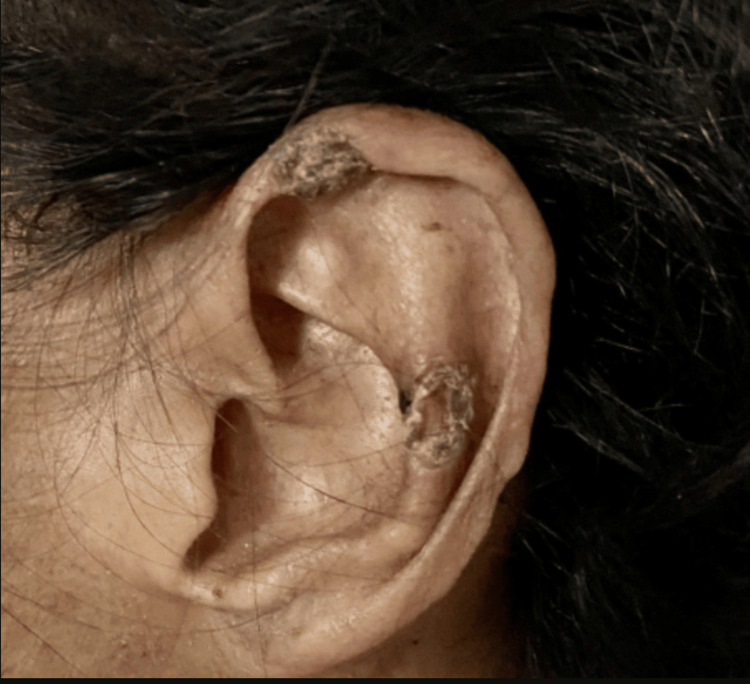
Discoid lupus-like plaques involving the helix of the ear Chronic hyperkeratotic plaques with crusting are observed on the auricular helix, consistent with long-standing discoid lupus–like cutaneous involvement

**Figure 2:**
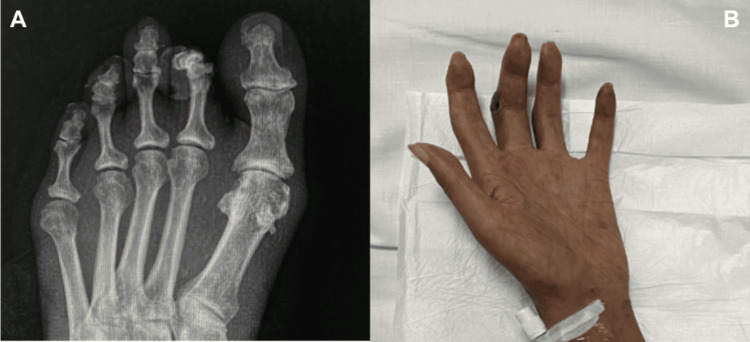
Musculoskeletal findings associated with mixed connective tissue disease (A) Foot radiograph demonstrating subchondral sclerosis, narrowing of the joint space, and marginal osteophyte formation. (B) Clinical photograph of the hand showing morphological changes consistent with sclerodactyly

At the time of admission to our service, the patient presented with progressive gangrenous lesions affecting the toes, which significantly limited her ability to walk. In addition, ulcerative lesions were observed on both hands. Physical examination revealed cyanotic facies with apparent loss of subcutaneous tissue and areas of alopecia. Discoid lupus-like plaques of long evolution were present on the helix of the ears with evidence of chronic skin damage (Figure 1). Radiographic evaluation showed subchondral sclerosis with reduction of the joint space and marginal osteophytes (Figure 2A). Musculoskeletal examination demonstrated morphological changes consistent with sclerodactyly of the hands (Figure 2B).

**Figure 3:**
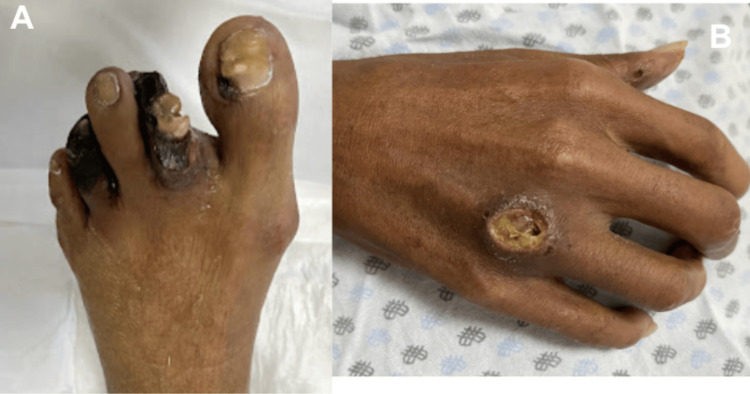
Ischemic and ulcerative cutaneous lesions associated with severe peripheral vascular involvement (A) Clinical photograph of the foot demonstrating extensive gangrenous and necrotic lesions affecting multiple toes, with devitalized tissue and purulent secretion. (B) Ulcerative lesion on the dorsum of the hand extending to the subcutaneous tissue, with erythematous borders and perilesional scaling

Examination of the lower extremities revealed extensive gangrenous and necrotic lesions involving the toes, with devitalized tissue, purulent secretion, and generalized loss of subcutaneous tissue (Figure 3A). Additionally, an ulcerative lesion extending to the subcutaneous tissue with erythematous borders and perilesional scaling was identified on the dorsum of the hand (Figure 3B).

Given the severity of the vascular compromise and tissue necrosis, a multidisciplinary approach involving angiology, rheumatology, and psychology services was implemented. Local wound care and supportive management were initiated; however, the lesions demonstrated a poor clinical evolution. Due to the progressive nature of the ischemic and necrotic lesions, definitive surgical management was planned, consisting of bilateral infracondylar disarticulation.

During hospitalization, the patient remained hemodynamically stable. However, several hours before the scheduled surgical intervention, she developed sudden cardiorespiratory arrest and died despite resuscitation efforts.

## Discussion

MCTD is a rare, systemic autoimmune disorder characterized by overlapping clinical features of systemic lupus erythematosus, systemic sclerosis, and inflammatory myopathies, together with anti-U1 ribonucleoprotein antibodies [1,2]. Because of its heterogeneous presentation and the absence of universally accepted diagnostic criteria, diagnosis may be challenging, particularly in patients with atypical or severe extra-organ manifestations. The present case is clinically relevant because it illustrates an uncommon and devastating vascular presentation of MCTD, namely severe peripheral arterial ischemia progressing to digital gangrene, in the context of advanced multisystem disease.

Although Raynaud’s phenomenon and digital vasculopathy are well-recognized vascular manifestations of MCTD, progression to extensive ischemia and gangrene is distinctly uncommon and suggests profound vascular injury. In this patient, the coexistence of Raynaud’s phenomenon, sclerodactyly, interstitial lung disease, and pulmonary arterial hypertension reflects a severe systemic phenotype. Pulmonary involvement, particularly interstitial lung disease and pulmonary arterial hypertension, is among the main determinants of morbidity and mortality in MCTD and often signals a more aggressive disease course [3]. In this context, the extensive peripheral ischemia observed in our patient may represent the vascular expression of advanced systemic autoimmune injury rather than an isolated local complication.

The pathophysiology underlying severe ischemic complications in MCTD is likely multifactorial. Increasing evidence supports a central role for endothelial dysfunction, chronic immune-mediated inflammation, and microvascular damage in disease progression. Elevated levels of markers associated with endothelial activation, including soluble intercellular adhesion molecule-1, interleukin-6, and von Willebrand factor, have been described in patients with MCTD, supporting the concept of persistent vascular injury as a key component of the disease [4]. Chronic inflammation, autoantibody-mediated immune activation, and progressive endothelial damage may together promote microvascular occlusion, impaired perfusion, and ultimately irreversible tissue ischemia. The severity of vascular compromise in the present case is consistent with this pathogenic framework.

The literature describing severe ischemic manifestations in MCTD remains limited, which further underscores the importance of reporting this case. While most vascular manifestations are confined to vasospasm or chronic digital vasculopathy, rare reports have documented more severe vascular occlusive events affecting other organ systems, including retinal arterial occlusions [5,6]. Collectively, these observations support the notion that, in susceptible patients, MCTD can progress beyond functional vasospasm to clinically significant vascular obstruction and tissue loss. Our case expands this spectrum by documenting rapidly progressive peripheral arterial ischemia with gangrenous involvement and fatal clinical deterioration.

From a clinical perspective, this case emphasizes the importance of maintaining a high index of suspicion for systemic autoimmune disease in patients presenting with unexplained ischemic or necrotic lesions, especially when accompanied by Raynaud’s phenomenon, sclerodactyly, inflammatory arthropathy, interstitial lung disease, or other findings suggestive of connective tissue disease. Early rheumatologic evaluation, including autoantibody testing and assessment for systemic organ involvement, is essential in such scenarios [7]. Multidisciplinary collaboration among rheumatology, internal medicine, vascular specialists, and surgical teams is likewise critical, particularly in patients with progressive tissue loss or limb-threatening ischemia.

Therapeutic management of MCTD remains largely manifestation-driven, as disease-specific treatment guidelines are limited. In general, treatment strategies are extrapolated from other connective tissue diseases and commonly include glucocorticoids, antimalarial agents such as hydroxychloroquine, and immunosuppressive therapies in cases with major organ involvement [3]. Nevertheless, as illustrated by this case, severe vascular complications may still occur despite supportive and multidisciplinary care, particularly when the disease is advanced or vascular injury is already extensive at presentation. This reinforces the need for earlier recognition of autoimmune vascular involvement before irreversible ischemic damage develops.

This case report has several strengths, including the documentation of a rare and severe vascular manifestation of MCTD and its correlation with a broader systemic autoimmune phenotype. By situating the patient’s presentation within the existing literature, this report contributes to expanding the current understanding of the disease spectrum and may improve recognition of similar presentations in future practice. At the same time, several limitations must be acknowledged. As a single case report, the findings cannot establish causality or be generalized to all patients with MCTD. The retrospective and descriptive nature of the report also limits the availability of some clinical details and introduces inherent selection and reporting bias. Moreover, the vascular complication described here may represent an extreme manifestation rather than a typical feature of the disease.

Despite these limitations, this report highlights an important clinical message: severe peripheral ischemia and digital gangrene may occur in MCTD as a consequence of advanced immune-mediated vascular injury and should not be overlooked in patients with compatible systemic features. Greater recognition of such presentations may facilitate earlier diagnosis, more timely immunomodulatory treatment, and more appropriate multidisciplinary management. Further research is needed to clarify the mechanisms linking autoimmune activation and vascular injury in MCTD, identify predictors of severe ischemic complications, and define optimal strategies for prevention and treatment.

## Conclusions

MCTD is a complex autoimmune disorder with a highly heterogeneous clinical spectrum that may involve multiple organ systems. Although vascular manifestations such as Raynaud’s phenomenon are common, severe peripheral ischemia leading to digital gangrene is a rare but potentially life-threatening complication. This report highlights the importance of maintaining a high index of suspicion for systemic autoimmune disease in patients presenting with unexplained peripheral ischemia or necrotic lesions. Early recognition, prompt immunologic evaluation, and multidisciplinary management are essential to prevent irreversible tissue damage and improve clinical outcomes. Further studies are needed to better understand the mechanisms underlying vascular injury in MCTD and to identify patients at risk of severe ischemic complications.
